# A water-stable lanthanide metal-organic framework for fluorimetric detection of ferric ions and tryptophan

**DOI:** 10.1007/s00604-017-2306-0

**Published:** 2017-06-13

**Authors:** Hani Nasser Abdelhamid, Antonio Bermejo-Gómez, Belén Martín-Matute, Xiaodong Zou

**Affiliations:** 1 Inorganic and Structural Chemistry and Berzelii Center EXSELENT on Porous Materials, Department of Materials and Environmental Chemistry, Stockholm University, -106 91 Stockholm, SE Sweden; 2 Department of Organic Chemistry and Berzelii Center EXSELENT on Porous Materials, Stockholm University, -10691 Stockholm, SE Sweden

**Keywords:** Lanthanum-organic frameworks, Luminescence, Metal ions detection, Amino acids, Sensing

## Abstract

The preparation of a highly water stable and porous lanthanide metal-organic framework (MOF) nanoparticles (denoted SUMOF-7II; SU refers to Stockholm University) is described. SUMOF-7II was synthesized starting from the tritopic linker of 2,4,6-tri-p-carboxyphenyl pyridine (H_3_L2) and La(III) as metal clusters. SUMOF-7II forms a stable dispersion and displays high fluorescence emission with small variation over the pH range of 6 to 12. Its fluorescence is selectively quenched by Fe(III) ions compared to other metal ions. The intensity of the fluorescene emission drops drops linearly in 16.6–167 μM Fe(III) concentration range, and Stern-Volmer plots are linear. The limit of detection (LOD) is 16.6 μM (at an S/*N* ratio of >3). This indicator probe can also be used for selective detection of tryptophan among several amino acids. Compared to the free linker H_3_L2, SUMOF-7II offers improved sensitivity and selectivity of the investigated species.

**Electronic supplementary material:**

The online version of this article (doi:10.1007/s00604-017-2306-0) contains supplementary material, which is available to authorized users.

**Graphical abstract:**
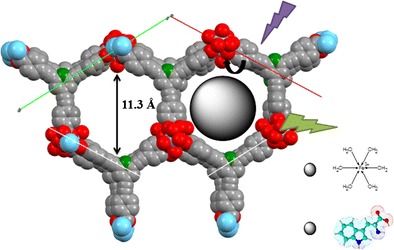
A water-stable porous lanthanide metal-organic framework SUMOF-7II (La) has shown to be an excellent probe for the detection of ferric ions among other metal ions, and tryptophan among other amino acids in aqueous solution. The new probe displays high and stable fluorescence signal in a wide pH range (6–12).

## Introduction

Ferric ion acts as an important metal center in catalysis and biotechnology, and plays a pivotal role in biology [1]. An appropriate level of Fe(III) intake/uptake prevents certain diseases such as heart, pancreas, Parkinson, Alzheimer and liver diseases [2]. However, when the concentration of free Fe(III) species exceeds the capacity of the organisms, they become harmful, although they can be detoxified via siderophores [2]. Therefore, the detection or sensing of Fe(III) is important for biomedical and environmental concerns [3]. Traditional analytical techniques such as inductively coupled plasma mass spectrometry (ICP-MS) provide accurate quantitative measurements of the metals. However, it lacks of selectivity and can only detect the total amount of Fe ions without discrimination among their different oxidation states (i.e. Fe(III) vs Fe(II)). They are expensive, time-consuming, and require pretreatment or preconcentration compared to other techniques such as luminescence spectroscopy [4, 5].

Amino acids play an important role in the biochemistry of mammalian cells [6]. Biosensing of amino acids has been investigated for UV-vis absorption [7], or electrochemical methods [8]. These methods show high sensitivity and selectivity toward amino acids. However, they require expensive chemicals such as enzyme, and long acquisition time. On the other hand, fluorescent biosensors are simple, sensitive and cheap [9], but it is hard to develop fluorescent biosensors for detection and recognition of individual amino acids due to the high similarity in their chemical structures [10].

Metal-organic frameworks (MOFs) constitute a group of attractive hybrid materials with interesting applications such as gas separation [11] and storage [12], extraction/preconcentration [13], sensing [14, 15], drug delivery [16], and catalysis [17]. They have also been explored for luminescence applications [18]. MOFs contain both organic and inorganic moieties, and have diverse structures and topologies [19]. They can offer sharp and clear emissions, high optical purity with high quantum yields and relatively long lifetime [20–24]. The luminescence of MOFs can be turned off by certain metal ions such as Fe(III) species, which is promising for chemosensing [25, 26]. Wei et al. reported that rigidified fluorescent linker effectively tunes the frontier orbital energy gap and thus improves the photoluminescence [19]. One drawback of organic molecules as fluorescence probes for metal ions is that they suffer from self-quenching and have low quantum yields. One solution to overcome this drawback is to incorporate the organic fluorophores as the linkers in a MOF to rigidify the molecules. There are two distinct advantages for such an approach. First, the linkers can adopt some special conformations that would otherwise be difficult; hence they may have different fluorescence or absorption energies [19]. Second, the linker in a MOF is separated from one another, which promotes photoluminescence and reduces self-quenching [19]. A desired fluorescence emission can be achieved by combining an optimum metal center and an effective organic linker. Lanthanide-based MOFs offers many advantages such as variable coordination geometries, multi-emission, and high structure stability. These advantages of MOFs compared to traditional probes advanced the applications of sensing and biosensing [15, 27].

A series of new isostructural lanthanide-based MOFs, SUMOF-7I to –IV using different tri-topic linkers (L1-L4) was reported [28]. The family of SUMOF-7 series exhibits high thermal and chemical stability. They are stable in water and organic solvents and in both acid and basic aqueous solutions. The materials show promising luminescence properties. SUMOF-7II(La) (denote SUMOF-7II) was selected to study its potential applications for sensing metal ions and amino acids. SUMOF-7II is built from La—O chains connected by 2,4,6-tri-*p*-carboxyphenylpyridine linkers (H_3_L2), and has large 1D channels (11.3 Å) that are accessible for small guest species. SUMOF-7II exhibits high selectivity and sensitivity for the detection of Fe(III) ions among many other metal ions. It can distinguish between different oxidation states of iron (Fe(II) and Fe(III)) and discriminate among solutions of various Fe(III) salts such as chlorides, acetate, nitrates and sulfates. It can also detect tryptophan among other selected amino acids.

## Experimental

All chemicals were purchased from Sigma Aldrich (www.sigmaaldrich.com/sweden.html) and used without any purification.

## Instrumentation

Fluorescence and UV-vis absorption spectra were recorded on Varian Cary Eclipse Fluorescence and Perkin Elmer spectrophotometer, respectively. The fluorescence spectrophotometer experiments were performed at room temperature with a photomultiplier voltage of 700 V, a scan speed of medium, an excitation slit width of 5 nm, and an emission slit width of 5 nm. The fluorescence emission spectra were recorded in the wavelength range of 300–800 nm upon excitation at 285 nm. Fourier transform infrared (FT-IR) spectra (4000–400 cm^−1^) were recorded on a Varian 610-IR FT-IR spectrometer (UK). Powder X-ray diffraction (PXRD) patterns were recorded on a PANanalytical X’Pert PRO diffractometer coupled with Cu K_α1_ radiation (λ = 1.5406 Å). Thermogravimetric analysis (TGA) was performed in air from 25 °C to 650 °C with a heating rate of 2 °C/min using thermogravimetric analyzer (Perkin Elmer TGA 7). Scanning electron microscopy (SEM) was performed on JEOL JSM-7000F and JEOL JSM-7401F at an accelerating voltage of 10.0 kV and 2 kV, respectively. The crystals were ground and dispersed in ethanol (10 mL), and then 10 μL was placed on a carbon film and dried before the analysis. The size distribution of the dispersed SUMOF-7II was estimated from the dynamic light scattering (DLS, Zetasizer nanoseries, Malvern, UK).

X-ray absorption spectroscopy (XAS) was performed at MAX IV Laboratory, Lund University, Sweden. The data was collected in fluorescence mode. The experiment was conducted at 3.0 GeV and 50 to 100 mA, with the use of a Si (220) double-crystal monochromator that was detuned by 50%, and a Fe foil for internal calibration with the first inflection point defined as 7111.2 eV. The XAS data were extracted using the software EXAFSPAK and plotted using Origin 9.

## Sample preparation for PXRD and XAS

A 30 mg of SUMOF-7II powder was dispersed in 2 mL of Fe(III) solutions (Fe(AcO)_3_ or FeCl_3_) with a high concentration (100 mM). The suspension was then centrifuged at 5000 rpm for 15 min to collect SUMOF-7II powder. After washing with deionized water, the SUMOF-7II powders were analyzed using PXRD and XAS.

## Synthesis and characterization of [La(L2)(H_2_O)]·solvent (SUMOF-7II)

SUMOF-7II was prepared according to our previously reported protocol [28]. A mixture of 2,4,6-tri-*p*-carboxyphenylpyridine (H_3_L2, Fig. S1a, 0.1 mmol, 43.0 mg), LaCl_3_·7H_2_O (0.1 mmol, 37.2 mg), *N,N*-dimethylformamide (DMF, 5 mL), cyclohexane (2.5 mL) and H_2_O (1.25 mL) was sealed in a 20 mL glass vial and heated at 85 °C for 16 h. The reaction mixture was slowly cooled down to room temperature. Yellow crystals of SUMOF-7II were isolated, washed with DMF (10 mL) and ethanol (2×10 mL).

## Preparation of SUMOF-7II suspension, H_3_L2 solution and stock solutions

A suspension of SUMOF-7II (2 mg**·**mL^−1^) was prepared by dispersing 20 mg of ground SUMOF-7II in ethanol (10 mL) followed by ultrasonication for 10 min. A solution of organic linker H_3_L2 (2 mg**·**mL^−1^) was prepared by dissolving 20 mg of H_3_L2 in DMF (10 mL).

Series of stock solutions (1.0 mM) for the metal ions were prepared in deionized water. This includes solutions of different Fe(III) salts, such as Fe(AcO)_3_, FeF_3_·3H_2_O, FeCl_3_·6H_2_O, Fe(NO_3_)_3_·9H_2_O, Fe_2_(SO_4_)_3_, metal salts such as Ca(NO_3_)_2_·3H_2_O, Mg(NO_3_)_2_·6H_2_O, Ni(NO_3_)_2_·6H_2_O, Zn(NO_3_)_2_·6H_2_O, LaCl_3_, AlCl_3_, NaF, FeSO_4_·7H_2_O, K_2_CO_3_, NaI, NaH_2_PO_4_, various acids and base HCl, HCOOH, AcOH, NaOH, and selected amino acids L-asparagine, L-glutamine, L-histidine, L-leucine, L-methionine, and L-tryptophan.

## UV-vis absorption and fluorescence spectroscopy

Analyte solutions of Fe(III) or tryptophan with various concentrations were prepared by using different volumes (5–1500 μL) of the stock solution (1 mM). SUMOF-7II suspension (50 μL) or H_3_L2 solution (50 μL) was added to the analyte solution, and the volume was completed to 3 mL using aqueous phosphate buffer saline (PBS, pH value of 7.4). The experiments were carried out at the excitation wavelength 285 nm. Four different Fe(III) salts; Fe(AcO)_3_, FeF_3_, FeCl_3_ and Fe(NO_3_)_3_ were investigated.

For the measurement of the selectivity, 500 μL of the stock solution from an analyte (1 mM) was mixed with 50 μL of SUMOF-7II suspension (2 mg**·**mL^−1^) or H_3_L2 solution (2 mg**·**mL^−1^). The mixture was completed to 3 mL using aqueous phosphate buffer saline (PBS, pH 7.4). The same procedure was followed for Fe(III) solutions of different salts using 200 μL instead of 500 μL of the stock solutions to investigate the sensitivity of SUMOF-7II to Fe(III) salts.

## Results and discussion

### Material characterization of SUMOF-7II nanoparticles

**Fig. 1:**
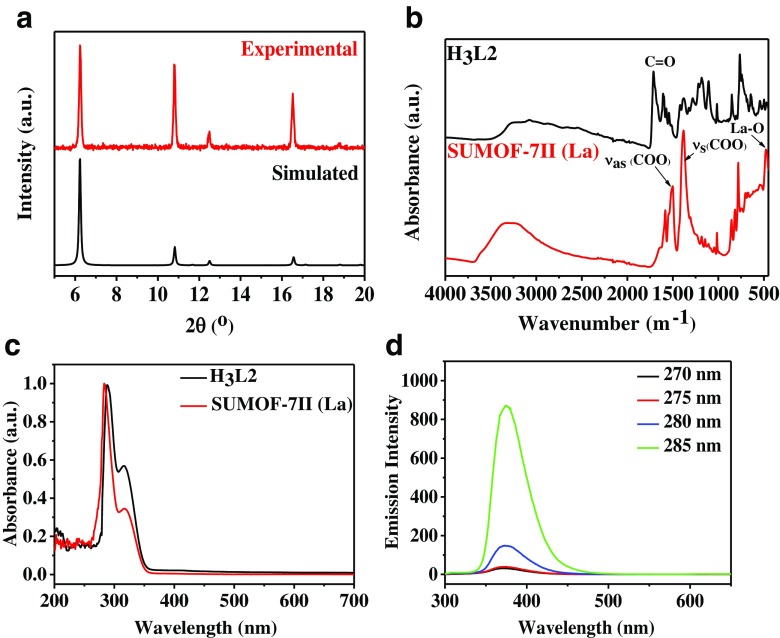
Characterization of SUMOF-7II, (**a**) Experimental (*top*) and simulated (*bottom*) PXRD patterns of SUMOF-7II, (**b**) FT-IR spectra and (**c**) UV-vis absorption spectra of SUMOF-7II and H_3_L2, and (**d**) Fluorescence emission spectra of SUMOF-7II with different excitation wavelengths

The phase purity of SUMOF-7II was confirmed by comparing the experimental and simulated PXRD patterns (Fig. 1a), and also from the morphology shown from SEM images (Fig. S2). FT-IR spectra (Fig. 1b) show peaks of the asymmetric (υ_as_ (COO)) and symmetric (υ_s_ (COO)) stretching at 1554 and 1364 cm^−1^, respectively that are formed due to coordination of the carboxylic groups with lanthanum (La) metal clusters [29]. The broad peak at 3450 cm^−1^ refers to O—H stretching of the coordinated or absorbed water. The peak at 480 cm^−1^ is assigned as La—O stretching. No peak corresponding to the C—O—H stretching of the free linkers at 1227 cm^−1^ is observed, which indicates there is no unreacted linkers inside the pores of SUMOF-7II. Results of DLS (Fig. S3) and SEM (Fig. S4) reveal that the particle sizes of SUMOF-7II are 40–80 nm with an average size of 60 nm.

### Luminescence properties of SUMOF-7II and H_3_L2

The suspension of SUMOF-7II displays two UV absorption peaks at 285 and 316 nm, whereas the linker of H_3_L2 shows maximum absorption peaks at 288 and 317 nm (Fig. 1c). Upon the excitation at wavelength 285 nm, SUMOF-7II shows a higher emission than H_3_L2 (Fig. S5). This enhancement may be due to increased rigidity of the organic moieties and ligand-to-metal charge transfer (antenna effect) [19]. The fluorescence emission of dispersed SUMOF-7II using different excitation wavelengths is shown in Figs. 1d and S6. The emission intensity increases with the increase of the excitation wavelength, and reaches the maximum at 285 nm (Fig. 1d). Further increase of the excitation wavelength leads to the saturation of the emission of SUMOF-7II (Fig. S6). The emission of SUMOF-7II produces a narrow peak with the maximum fluorescence emission at 375 nm (Fig. 1d), and a large Stokes shift (excitation wavelength λ_ex_ of 285 nm, emission wavelength λ_em_ of 375 nm, Stokes shift of 90 nm). This data implies that the emission of SUMOF-7II can be tuned for biological applications where auto-fluorescence causes interference [30]. The fluorescence signal is nearly constant over a wide pH range of 6–12 (Fig. S7). The fluorescence emission signals show a small change for different synthesis batches (Fig. S8). This is because the fluorescence signals are due to ligand-to-metal charge transfer (antenna effect). The fluorescence emission of SUMOF-7II dispersed in ethanol remained unchanged during at least three months, which reveals the high photostability of SUMOF-7II (Fig. S9).

The high dispersion of SUMOF-7II together with its large pore size (11.3 Å) can enhance MOF-guest interactions for small analyte species. The potential applications of SUMOF-7II as a sensor to metal ions and amino acids were investigated.

### Selectivity and sensitivity of SUMOF-7II towards Fe(III) ions

**Fig. 2:**
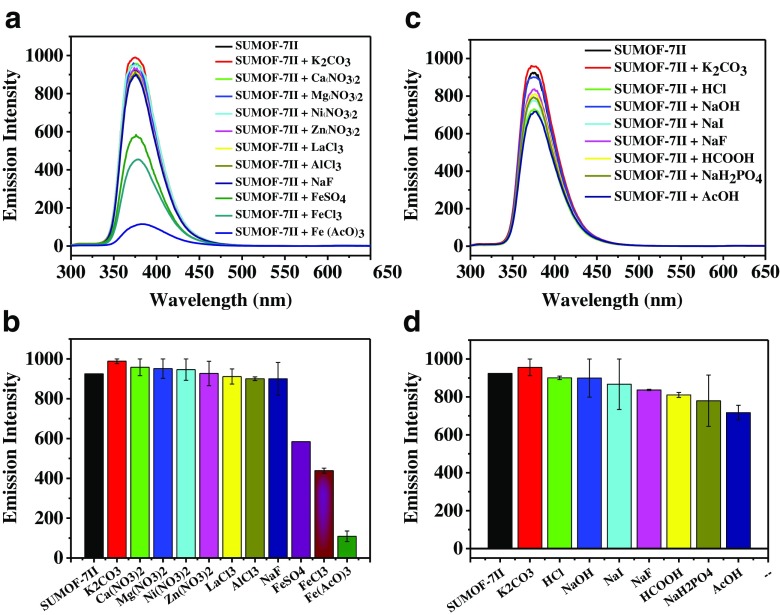
Fluorescence response of SUMOF-7II (0.03 mg·mL^−1^) to different metal salts (**a**, **c**) and different anion species (**b**, **d**) at the excitation wavelength of 285 nm. The concentration of the analyte solutions were 167 μM

To explore the potential of SUMOF-7II for detection of metal ions, the responses of the fluorescence emission of SUMOF-7II to different cations (Fig. 2a) and anions (Fig. 2b) were investigated. SUMOF-7II only shows significant responses to Fe(II) and Fe(III) among the tested metal ions (Fig. 2a). The presence of Fe(III) ions (FeCl_3_ or Fe(AcO)_3_) drastically decreases the fluorescence emission of SUMOF-7II. Aqueous Fe(II) species also show fluorescence quench on SUMOF-7II. This is due to the presence of Fe(III) impurities caused by auto-oxidation of Fe(II). The quenching of SUMOF-7II due to Fe(III) reveals that the fluorescence emission of SUMOF-7II can be selectively turned off in the presence of Fe(III). SUMOF-7II has a higher selectivity for Fe(III) compared to Fe(II). Significant differences in the response to different Fe(III) salts are observed. The solution of ferric acetate (Fe(AcO)_3_) exhibits a higher quenching effect compared to the solution of ferric chloride (FeCl_3_). This is because acetate ions (AcO^−^) contribute to the quenching (Fig. 2d). All other tested anions do not make significant changes in the fluorescence emission of SUMOF-7II. The interaction of various metal ions with SUMOF-7II was studied using UV-vis absorption (Fig. S10). The changes of UV-vis absorption of SUMOF-7II are due to the interaction of the metal ions with SUMOF-7II. In summary, SUMOF-7II shows high selectivity to Fe(III) ions.

**Fig. 3:**
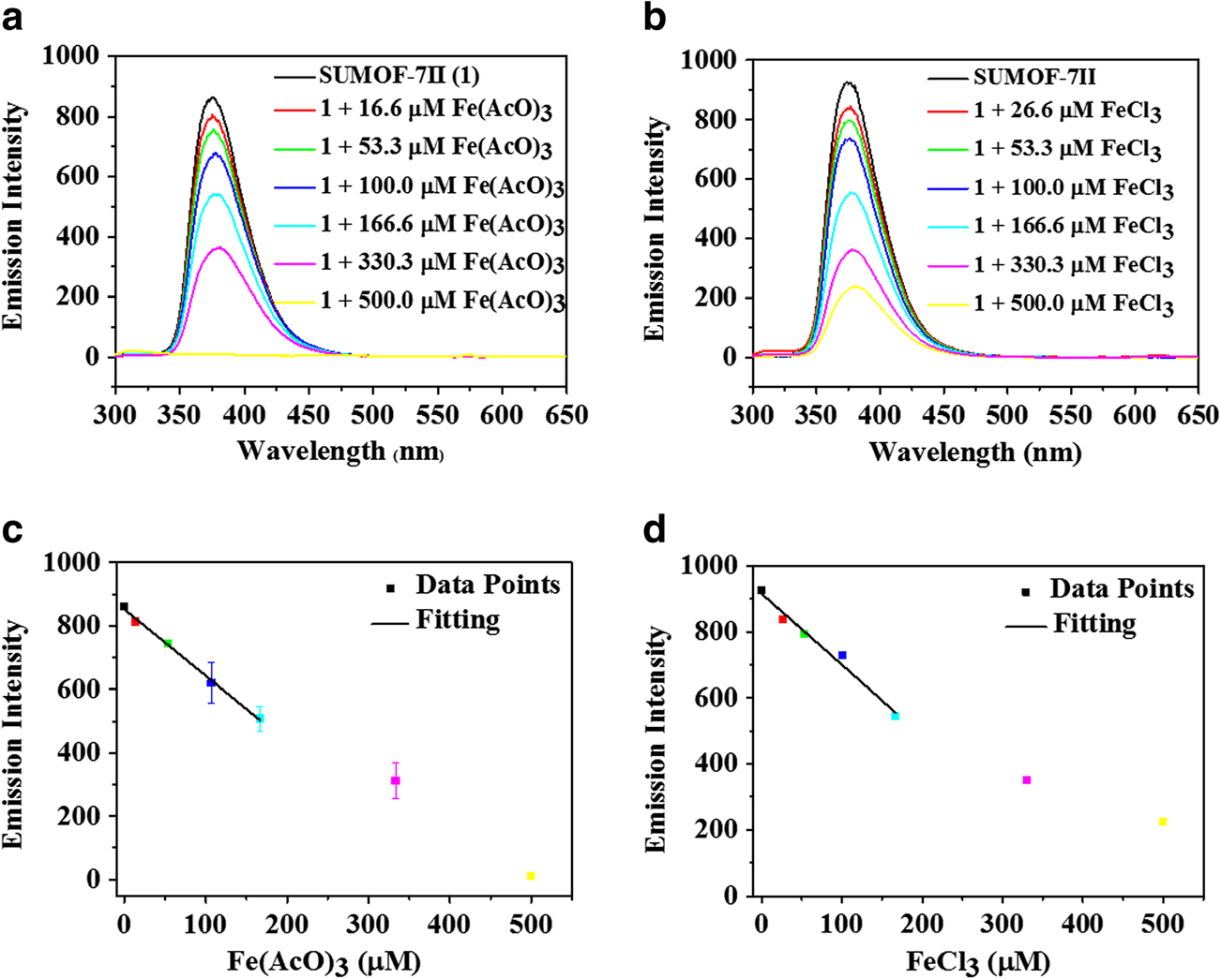
Fluorescence response of SUMOF-7II (0.03 mg·mL^−1^) upon addition of Fe(AcO)_3_ (**a**) and FeCl_3_ (**c**) at pH value of 7.4 (λ_ex_ = 285 nm), and as a function of the Fe(III) concentration for Fe(AcO)_3_ (**b**) and FeCl_3_ (**d**)

The sensitivity of SUMOF-7II towards FeCl_3_ and Fe(AcO)_3_ was investigated using fluorescence spectroscopy and UV-Vis absorption spectroscopy (Figs. 3a-c). The fluorescence emission of SUMOF-7II decreases with the increase of the Fe(III) concentration as shown in Fig. 3a for Fe(AcO)_3_ and Fig. 3c for FeCl_3_. The fluorescence signal shows a linear relationship with the concentration of Fe(III) in the range of 16.6–167 μM for Fe(AcO)_3_ (Fig. 3b, R^2^ = 0.99) and 26.6–167 μM for FeCl_3_ (Fig. 3d, R^2^ = 0.99). The quenching rate (QR) (QR = (I_0_ − I)/I_0_, where I_0_ and I are the fluorescence intensities of SUMOF-7II with and without the presence of Fe(III), respectively) does not change much with time and depends on Fe(III) salts (Fig. S11). This indicates that SUMOF-7II can be used to discriminate among these salts. The Stern-Volmer quenching constant (K_sv_), calculated using the Stern-Volmer equation (I_0_/*I* = 1 + K_sv_[Q], where [Q] represents the Fe(III) concentration), is 4.3 × 10^3^ M^−1^ for Fe(AcO)_3_ and 2.1 × 10^4^ M^−1^ for FeCl_3_ (Fig. S12). These values reveal a high quenching capability of Fe(III) ions due to the dynamical quenching. In addition, the fluorescence response of SUMOF-7II to Fe(III) is very fast (< 2 min), more than 5 times faster than that of MIL-53(Al) (> 10 min, MIL refers to Materials Institute Lavoisier) caused by cation exchange^17^. The analytical parameters of limit of detection (LOD), limit of quantification (LOQ) and linear range are tabulated in Table 1.

**Table 1:** Analytical parameters of Fe(III) for SUMOF-7II and H_3_L2

Probe	Analyte	LOD (μM)	Linear Range (μM)	R^2^	LOQ (μM)
SUMOF-7II	Fe(AcO)_3_	16.6	16.6–167	0.99	16.6
	FeCl_3_	26.6	26.6–167	0.99	26.6
	Tryptophan	167	167–500	0.98	167
H_3_L2	FeCl_3_	26.6	26.6–167	0.99	26.6
	Tryptophan	26.6	26.6–500	0.98	26.6

*LOD* limit of detection, *LOQ* limit of quantification

### Selectivity of SUMOF-7II towards amino acids

**Fig. 4:**
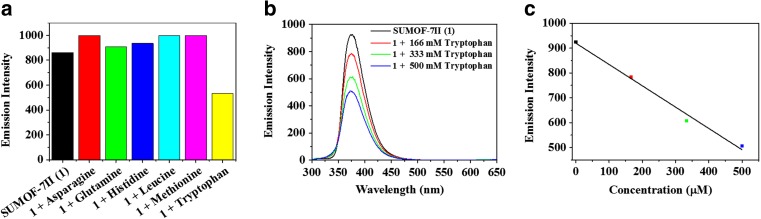
The fluorescence response of SUMOF-7II (**a**) in the presence of selected amino acids (167 μM) showing the selectivity of SUMOF-7II towards tryptophan and (**b**) at different concentrations of tryptophan and c) linear relationship.

The selectivity of SUMOF-7II towards selected amino acids, whose absorption or emission matches with the absorption or emission of SUMOF-7II, such as L-histidine, L-asparagine, L-glutamine, L-leucine, L-methionine and L-tryptophan were tested (Figs. 4 and S13). While other amino acids enhance the fluorescence emission of SUMOF-7II, only tryptophan causes selective quenching of SUMOF-7II (Fig. 4a). The fluorescence emission of SUMOF-7II shows a linear response with the concentration of tryptophan (Fig. 4b), which agrees with the Stern Volmer equation and gives a K_sv_ constant of 1.69 × 10^3^ M^−1^ (Fig. S14). The high selectivity of SUMOF-7II toward tryptophan is due to dynamic quenching and energy transfer. It is important to stress that both SUMOF-7II and tryptophan have similar absorbance at 285 nm. Thus, tryptophan causes selective quenching to the emission intensity of SUMOF-7II.

**Fig. 5:**
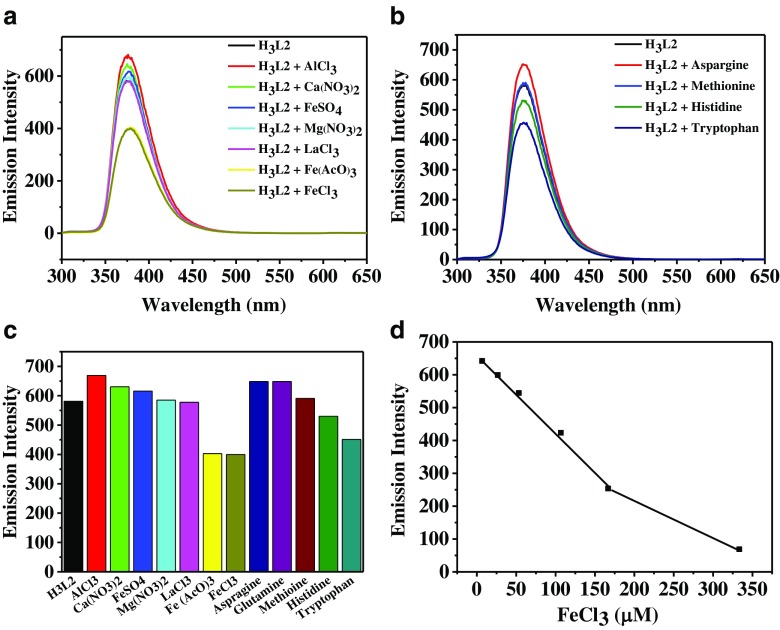
Fluorescence response of H_3_L2 (0.03 mg·mL^−1^) to various a) metal ions and b) amino acids (λ_ex_ = 285 nm, analytes concentration of 167 μM for metal ions and amino acids). c) Comparison of fluorescence responses of H_3_L2 towards various metal ions and amino acids. d) Fluorescence response of H_3_L2 towards FeCl_3_ as a function of the FeCl_3_ concentration

Because the linker H_3_L2 is the energy receptor and the source of fluorescence emission of SUMOF-7II, we also studied the fluorescence emission of H_3_L2 towards metal ions and selected amino acids (Fig. 5). H_3_L2 offers selectivity towards Fe(III) among the selected metal ions (Fig. 5a). However, SUMOF-7II offers significantly higher sensitivity than H_3_L2 (Table 1). For example, the quenching rate for Fe(AcO)_3_ is ~89% for SUMOF-7II and ~34% for H_3_L2 (Figs. 2b and 5c). Furthermore, different from SUMOF-7II, H_3_L2 does not show any selectivity between Fe(AcO)_3_ and FeCl_3_ (Fig. 5a and c). SUMOF-7II shows a wider linear concentration range of the fluorescence emission towards FeCl_3_ (Figs. 3d and 5d). H_3_L2 only shows a slightly high selectivity towards tryptophan among the selected amino acids (Fig. 5b).

### Characterization of SUMOF-7II after interactions with Fe(III) species

**Fig. 6:**
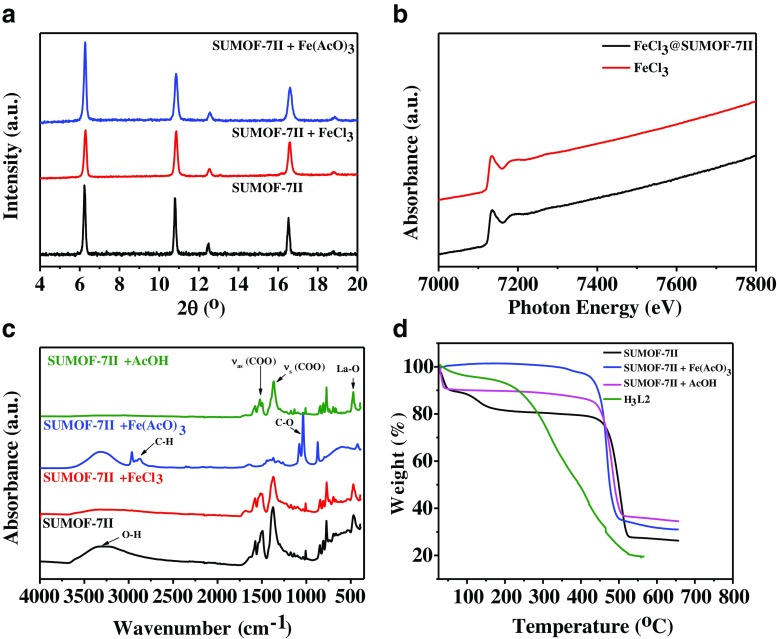
(**a**) PXRD of SUMOF-7II without and with FeCl_3_ or Fe(AcO)_3_, (**b**) XAS spectrum of Fe K-edge (E = 7112 eV) of FeCl_3_@SUMOF-7II, (**c**) FT-IR spectra of the interactions between SUMOF-7II and AcOH, FeCl_3_ and Fe(AcO)_3_, and (d) TGA curves

Powder X-ray diffraction shows that the crystallinity of SUMOF-7II is retained in the presence of Fe(III) salts (Fig. 6a). X-ray absorption spectroscopy shows that the Fe *K*-edge (7.112 keV) of FeCl_3_ and FeCl_3_@SUMOF-7II are the same (Fig. 6b), which indicates that the oxidation state of iron (+3) is remained in FeCl_3_@SUMOF-7II. A strong interaction of Fe(AcO)_3_ with SUMOF-7II is observed from the FT-IR spectra in Fig. 6c, indicating that Fe(AcO)_3_ plays an important role in quenching SUMOF-7II compared to other Fe(III) species. The peaks at wavenumber of 2990 and 1100 cm^−1^ refer to C—H and C—O from acetate and SUMOF-7II, respectively. The peak of La—O shifted from 480 cm^−1^ to 450 cm^−1^ in the presence of acetate ions (AcO^−^). The interaction with acetic acid (AcOH) was recorded as a control experiment (Fig. 6c). The peak splitting of (COO)_as_ indicates that acetate (AcO^−^) coordinated to the framework metal La (III). SUMOF-7II shows higher thermal stability (up to 500 °C) compared to the organic linker H_3_L2 (200 °C), although the adsorption of Fe(AcO)_3_ or AcOH causes a slight decrease of the stability (Fig. 6d). The high thermal stability of SUMOF-7II is important for storage, transportation and sensing at high temperatures.

It is important to mention that conventional organic fluorophores are usually insoluble in water (for instance, H_3_L2 is soluble in DMF). In contrast, SUMOF-7II is well-dispersed in ethanol and forms a stable suspension over a long time period (Fig. S9). SUMOF-7II shows a narrow peak (350–425 nm) compared to organic fluorophore. This feature is attributed to the low flexibility of the organic linker inside MOFs. SUMOF-7II offers ultra-sensitivity and shows a good linear relationship in concentration range 16.6–167 μM (Table 2). SUMOF-7II can be used for quantification and qualitative analysis with higher selectivity and better sensitivity compared to other probes (Table 2). It was performed in aqueous solution (pH = 7) without the requirement of acidic solution (pH = 3) [31]. SUMOF-7II offers a wide linear range compared to other probes such as boron doped carbon dots (B-CDs) [32]. It also requires no chelating agents [33]. SUMOF-7II can be applied in a wide pH range of 6–12, requires low concentration of the probe and shows fast response time (Table 2).

**Table 2:** An overview on reported nanomaterial-based methods for fluorometric determination of ferric ion

Fluorophore	Probe conc. (mg·mL^−1^)	LOD (μM)	Linear range (μM)	Response time (min)	pH range	Ref.
CPDs	0.05	0.1	0.2–10	30	3	[31]
B-CDs	0.5	0.242	0–16	1	ND	[32]
NA-GQDs	ND	0.1	0.5–500	5	7	[33]
MIL-53(Al)	0.05	0.90	3–200	7	4–10	[20]
Eu-MOF	4	ND	ND	> 320	7	[23]
Eu-MOF	1	0.33	ND	30	ND	[22]
UMCM-1-NH_2_	0.2	1000	ND	ND	ND	[24]
SUMOF-7II	0.03	16.6	16.6–167	< 1	6–12	Here
H_3_L2	0.03	26.6	26.6–167	< 1	7.4	Here

ND not detected, Carbon polymer dots, CPDs, Boron doped carbon dots, B-CDs Nitrogen-doped and amino acid functionalized graphene quantum dots, NA-GQDs UMCM-1-NH_2_, University of Michigan Crystalline Material-1

## Conclusions

A highly stable and porous lanthanide metal-organic framework nanoparticle (SUMOF-7II) was synthesized using simple grinding and ultrasonication of synthesized SUMOF-7II. Aqueous Fe(III) ions and tryptophan show selective quenching of the fluorescence emission of SUMOF-7II. The suspension of SUMOF-7II nanoparticles (~ 60 nm on average) shows a narrower peak (350–425 nm) with higher emission signals compared to organic fluorophore (H_3_L2). SUMOF-7II (La) offers a direct and label free method for the detection of Fe(III) and tryptophan. SUMOF-7II presents a new platform for future sensing and biosensing applications. More efforts are required to further increase the sensitivity and selectivity of the material.

## Electronic supplementary material

ESM 1 (DOC 2.34 MB)
