# Supplementary material for: A water-stable lanthanide metal-organic framework for fluorimetric detection of ferric ions and tryptophan
Source: Mikrochim Acta. 2017 Jun 13;184(9):3363–71. doi: 10.1007/s00604-017-2306-0 (PMC5552832; doi:10.1007/s00604-017-2306-0)
Supplement: Supplementary file 1 — (DOC 2.34 MB) [file 604_2017_2306_MOESM1_ESM.doc]

**Electronic Supplementary Material**

**A water-stable lanthanide metal-organic framework for fluorimetric detection of ferric ions and tryptophan**

Hani Nasser Abdelhamid^†^,*Antonio Bermejo-Gómez,^‡^ Belén Martín-Matute,^‡^ Xiaodong Zou^†^,*

^†^Inorganic and Structural Chemistry and Berzelii Center EXSELENT on Porous Materials, Department of Materials and Environmental Chemistry, Stockholm University, SE-106 91Stockholm, Sweden

^‡^Department of Organic Chemistry and Berzelii Center EXSELENT on Porous Materials, Stockholm University, SE-106 91Stockholm, Sweden

Email: hani.nasser@mmk.su.se (H.N.Abdelhamid); xzou@mmk.su.se (X.Zou)


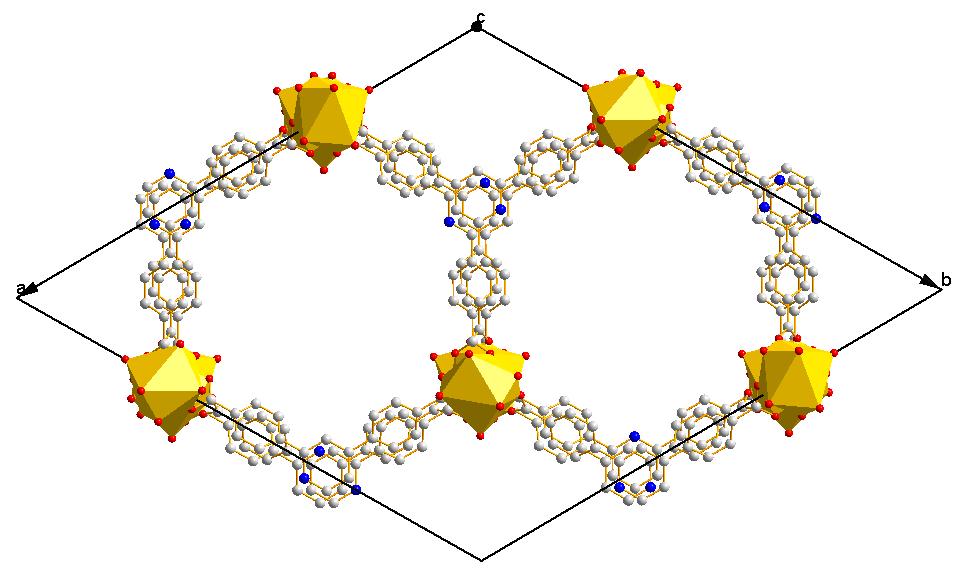


**a**

**b**

2,4,6-tri-p-carboxyphenylpyridine (H_3_L2) SUMOF-7II

**Figure S1.** (a) Chemical structure of the organic linker (H_3_L2) and (b) the 3D crystal structure of SUMOF-7II (*R*32, *a* = 28.320(5) Å, *c* = 12.835(3) Å). SUMOF-7II is built from La-O chains connected by the L2 linkers to form 1D channels with pore opening of 11.3 Å (after subtracting the van der Waals radii of atoms).


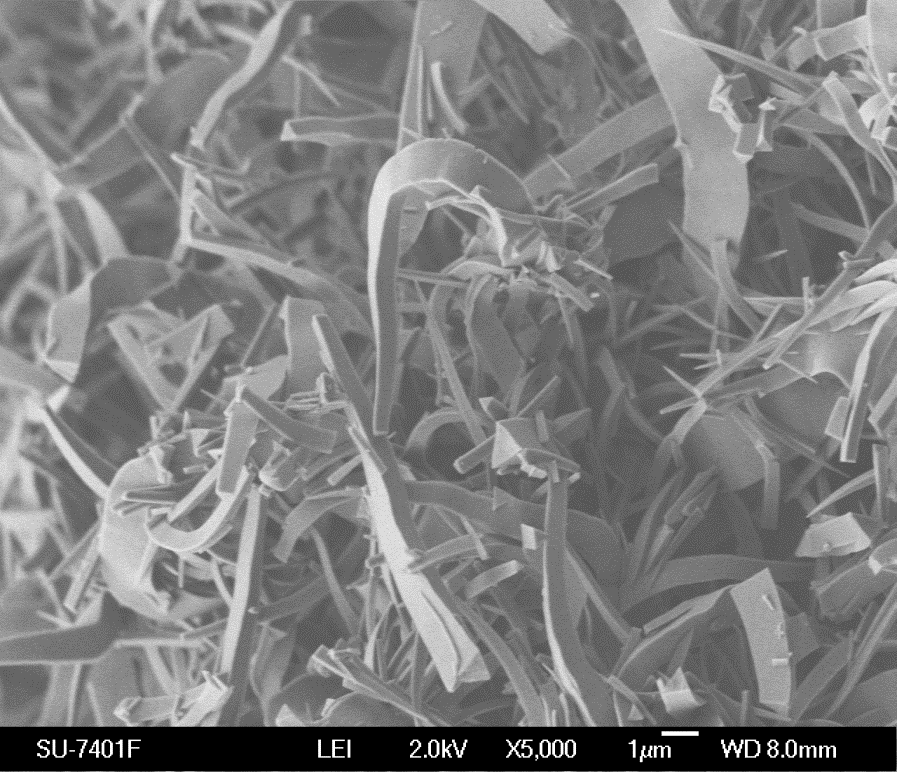


**Figure S2.** SEM image of SUMOF-7II.

**Figure S3.** DLS analysis of ground SUMOF-7II dispersed in ethanol (95 %).


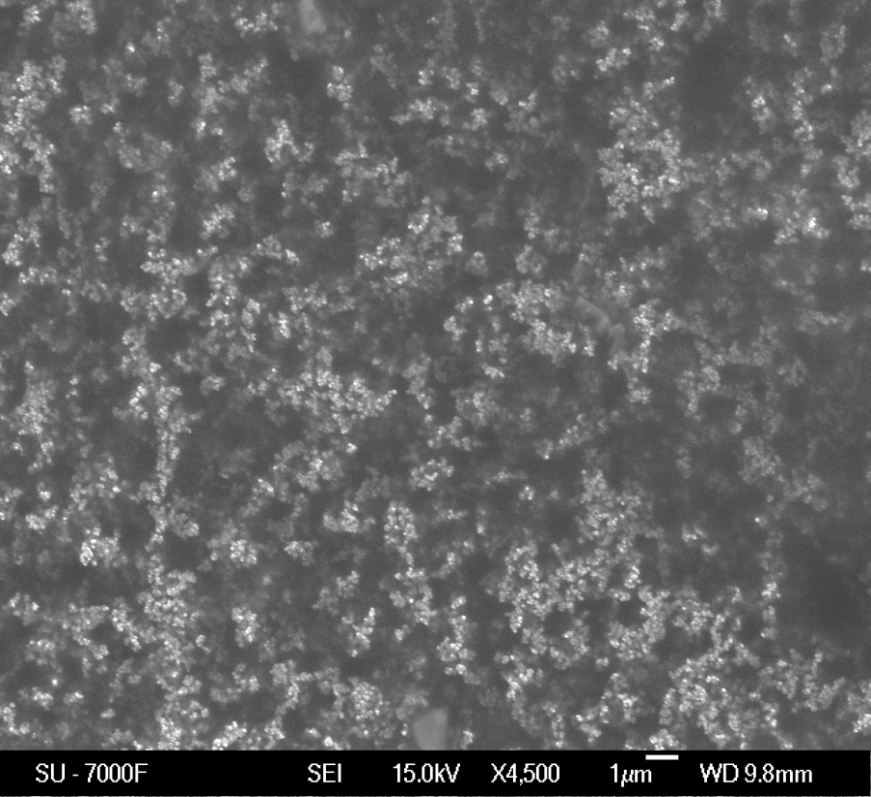


**Figure S4.** SEM image of ground SUMOF-7II dispersed in ethanol (95%).

**Figure S5**. Fluorescence emission of SUMOF-7II and H_3_L2 linker dispersed in ethanol (0.03 mg/mL) at excitation wavelength 285 nm, pH value 7.4.

**Figure S6.** Effect of the excitation wavelength on the emission of SUMOF-7II.

**Figure S7.** Effect of pH on the emission of SUMOF-7II dispersed in ethanol (0.03 mg·mL^-1^) at excitation wavelength 285 nm.

**Figure S8.** The emission of different SUMOF-7II batches dispersed in ethanol (0.03 mg·mL^-1^) at excitation wavelength 285 nm.

**Figure S9.** Fluorescence emission of SUMOF-7II dispersed in ethanol (0.03 mg·mL^-1^) with the time, showing the high photostability (λ_ex_= 285 nm).

**Figure S10**. UV-vis absorption spectra of SUMOF-7II in solutions of different metal ions.

**Figure S11.** Quenching rate of SUMOF-7II as a function of time in 167 μM Fe^3+^ solution of Fe(AcO_3_), FeCl_3_, Fe(NO_3_), and FeF_3_, respectively.

**a**

**
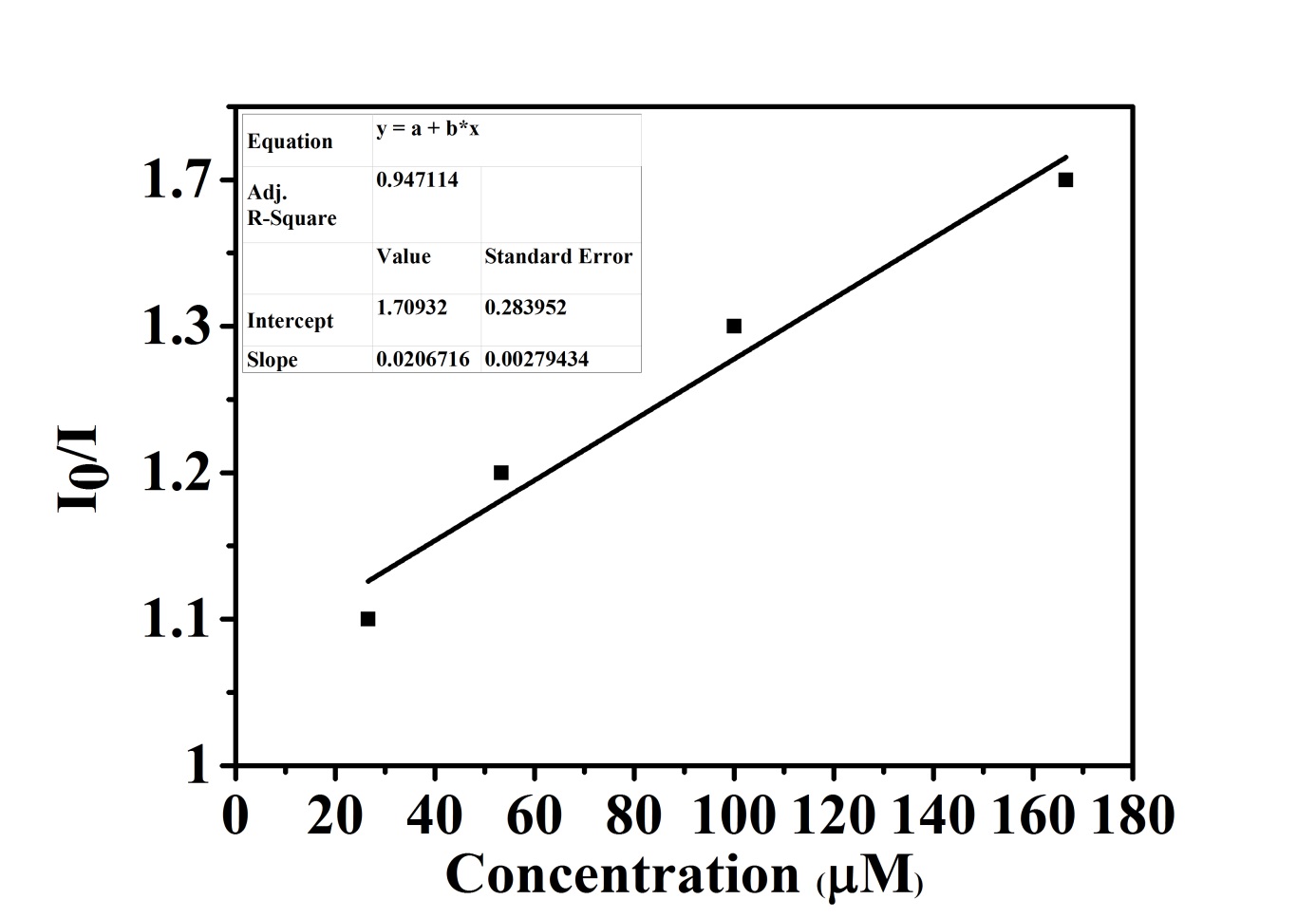
**

**b**

**Figure S12**. Fluorescence response of SUMOF-7II as a function of the concentration for (a) Fe(AcO)_3_ and (b) FeCl_3_.

**Figure S13.** Fluorescence emission of selected amino acids and the response of SUMOF-7II with those amino acids.

**Figure S14.** Linear fitting of fluorescence response I_0_/I of SUMOF-7II as a function of the tryptophan concentration showing that it follows the Stern Volmer equation.
